# Emerging roles of extracellular vesicles in COVID‐19, a double‐edged sword?

**DOI:** 10.1111/imm.13329

**Published:** 2021-05-04

**Authors:** Xiaohuan Xia, Ping Yuan, Yihan Liu, Yi Wang, Weijun Cao, Jialin C. Zheng

**Affiliations:** ^1^ Center for Translational Neurodegeneration and Regenerative Therapy Shanghai Tenth People’s Hospital Affiliated to Tongji University School of Medicine Shanghai China; ^2^ Translational Research Institute of Brain and Brain‐Like Intelligence Shanghai Fourth People's Hospital Affiliated to Tongji University School of Medicine Shanghai China; ^3^ Department of Cardio‐Pulmonary Circulation, Shanghai Pulmonary Hospital Tongji University School of Medicine Shanghai China; ^4^ Collaborative Innovation Center for Brain Science Tongji University Shanghai China

**Keywords:** COVID‐19, Extracellular vesicle, SARS‐CoV‐2, Cytokine storm, Inflammation

## Abstract

The sudden outbreak of SARS‐CoV‐2‐infected disease (COVID‐19), initiated from Wuhan, China, has rapidly grown into a global pandemic. Emerging evidence has implicated extracellular vesicles (EVs), a key intercellular communicator, in the pathogenesis and treatment of COVID‐19. In the pathogenesis of COVID‐19, cells that express ACE2 and CD9 can transfer these viral receptors to other cells via EVs, making recipient cells more susceptible for SARS‐CoV‐2 infection. Once infected, cells release EVs packaged with viral particles that further facilitate viral spreading and immune evasion, aggravating COVID‐19 and its complications. In contrast, EVs derived from stem cells, especially mesenchymal stromal/stem cells, alleviate severe inflammation (cytokine storm) and repair damaged lung cells in COVID‐19 by delivery of anti‐inflammatory molecules. These therapeutic beneficial EVs can also be engineered into drug delivery platforms or vaccines to fight against COVID‐19. Therefore, EVs from diverse sources exhibit distinct effects in regulating viral infection, immune response, and tissue damage/repair, functioning as a double‐edged sword in COVID‐19. Here, we summarize the recent progress in understanding the pathological roles of EVs in COVID‐19. A comprehensive discussion of the therapeutic effects/potentials of EVs is also provided.

## Graphical abstract

Extracellular vesicles (EVs) are a novel mediator of COVID‐19 pathogenesis/treatment. EVs facilitate viral spreading via transferring viral particles and receptors to recipient cells. Stem cell‐derived EVs can be used as therapeutic “drug” to treatment COVID‐19 or drug delivery platform to fight against COVID‐19.

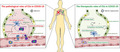

**Abbreviations**
ACE2: angiotensin‐converting enzyme 2
Alix: ALG‐2‐interacting protein X
ARF6: adenosine diphosphate ribosylation factor 6
ASMCs: airway smooth muscle cells
BALF: bronchoalveolar lavage fluid
BUN: blood urea nitrogen
COV: coronavirus
COVID‐19: coronavirus disease 2019
CQ: chloroquine
DC‐SIGN: Dendritic Cell‐Specific Intercellular adhesion molecule‐3‐Grabbing Non‐integrin
DIC: disseminated intravascular coagulation
EBV: Epstein–Barr virus
ECM: extracellular matrix
EEs: early endosomes
ESCRT: endosomal sorting complexes required for transport
EVs: extracellular vesicles
fMLP: formyl‐methionine‐leucine‐phenylalanine
HCQ: hydroxychloroquine
HIV: human immunodeficiency virus
i.t.: intratracheal instillation
ICU: intensive care unit
IFN: interferon
IL‐1: interleukin 1
IL‐6: interleukin 6
IL‐8: interleukin 8
ILVs: intraluminal vesicles
KC: keratinocyte‐derived cytokine
KGF: keratinocyte growth factor
LMP1: latent membrane protein 1
LPS: lipopolysaccharide
MCP1: monocyte chemoattractant protein 1
MERS‐CoV: Middle East respiratory syndrome coronavirus
MHC: major histocompatibility complex
MIP‐2: macrophage inflammatory protein 2
miRNA: MicroRNA
MODS: multiple organ dysfunction syndrome
MSC‐EVs: MSC‐derived EVs
MSCs: mesenchymal stromal/stem cells
MSOF: multi‐system organ failure
MVBs: multivesicular bodies
MVs: microvesicles
NE: neutrophil elastase
NSP: nonstructural proteins
NTA: nanotracking analysis
ORF: open reading frames
P2X7: P2X ligand‐gated ion channel 7 (P2X7)
PD: peptidase domain
PEF: pulmonary oedema fluid
PMEL: premelanosome protein
ROS: reactive oxygen species
SARS‐CoV: severe acute respiratory syndrome coronavirus
SARS‐CoV‐2: severe acute respiratory syndrome coronavirus 2
SIRS: systemic inflammatory response syndrome
TMPRSS2: transmembrane protease serine type 2
TNF‐α: tumour necrosis factor‐alpha
Tsg101: tumour susceptibility gene 101
TxA2: thromboxane A2
Vamp3: vesicle‐associated membrane protein 3
VWF: von Willebrand factor

## INTRODUCTION

Since December 2019, a novel coronavirus infectious disease has been prevalent in Wuhan, China, and has rapidly spread globally to become a pandemic [1]. This disease, caused by the infection of severe acute respiratory syndrome coronavirus 2 (SARS‐CoV‐2), is named as coronavirus disease 2019 (COVID‐19) by World Health Organization (WHO). COVID‐19 has seriously affected the quality of life, which further causes great economic, social and family burden. Understanding the molecular mechanisms underlying the pathogenesis of COVID‐19 from the SARS‐CoV‐2 infection to cytokine storm‐mediated organ failure will provide us with extremely valuable information to overcome COVID‐19. Extracellular vesicles (EVs), the nanoscale bilayer vesicles that can be released by virtually all eukaryotic cells, have emerged as an essential intercellular communicator [2, 3]. By horizontally transferring biomolecules like nucleic acids, proteins and lipids, EVs can regulate physiological and pathological processes of the recipient cells [2, 4, 5, 6]. Mounting evidence has implicated EVs in the pathogenesis and treatment of various lung diseases including COVID‐19 [3, 7]. For example, primed macrophage‐derived EVs facilitate the influx of inflammatory cells into the lung, leading to cytokine storms and acute lung injury [8]. In this review, we firstly discuss the epidemiology, symptoms and pathogenesis of COVID‐19 and its complications, together with the biogenesis, composition and origins of lung EVs. We then provide a comprehensive summarization for the roles of EVs in the infection of SARS‐CoV‐2, the progression of cytokine storm and the treatment of COVID‐19.

## COVID‐19 AND SARS‐COV‐2

### Epidemiology of COVID‐19

COVID‐19, caused by SARS‐CoV‐2, has spread globally and been accepted as a pandemic after initially occurring in China in December 2019. Till February 13th 2021, a total of 107,423,526 cases of COVID‐19 have been confirmed worldwide including 2,360,280 deaths, based on the record released from WHO (https://covid19.who.int/). The epidemic situation in China has been controlled except for sporadic cases. Unfortunately, new cases are being reported at increasing rates globally, and 83 countries, territories and areas had more than 100,000 cases. The largest number of confirmed cases is from the United States to this date. Given the severity of COVID‐19, it is urgently needed to develop therapeutics or vaccines to treat or prevent the pandemic. Until now, there are no effective drugs to treat COVID‐19 [9]. Encouragingly, based on WHO’s record on February 13th 2021, more than 60 COVID‐19 vaccine candidates are in clinical development, which may save millions of lives in the COVID‐19 pandemic crisis.

SARS‐CoV‐2 is spreading rapidly through different routes including droplets and close contact [10]. Among various viral transmission modes, the weight, from large to small, may be droplets, aerosol, fomite and faecal–oral route transmission. The majority of COVID‐19 cases develop a respiratory tract infection. Then, SARS‐CoV‐2 spreads by means of the respiratory tract, via droplets and respiratory secretions through coughing/sneezing and/or direct contact with infected individual. SARS‐CoV‐2 survives for several hours/days on different surfaces and persists in the air after an aerosolization process for distant transmission (over two metres), which further increases its infection risk [11]. Moreover, SARS‐CoV‐2 can be spread from asymptomatic cases, minimally symptomatic patients with high titres of viral load on pharyngeal samples during the initial days of the disease, and even patients on clinical recovery [12]. Therefore, densely populated areas or hospitals are the places with the highest viral transmission rate. Healthy and immunosuppressed population are similarly susceptible upon exposure to SARS‐CoV‐2 [12, 13]. The median age of patients was 59 years ranging from 15 to 89 years, and more than half of them were males [13]. People with low immune function particularly the elderly and those with renal and hepatic dysfunction are a higher risk group for severe COVID‐19 [14, 15]. Children have a milder disease course and better prognosis than adults [13, 16].

### Symptoms of COVID‐19

SARS‐CoV‐2 infects the lower airway respiratory tract and causes pneumonia that appears milder than severe acute respiratory syndrome coronavirus (SARS‐CoV) or Middle East respiratory syndrome coronavirus (MERS‐CoV) infection [17]. But it may lead to fatal acute lung injury, acute respiratory distress syndrome (ARDS), septic shock and multiple organ dysfunction syndrome (MODS) in nearly 10%–20% of the cases, resulting in death within a short time [17].

The time period from onset of COVID‐19 symptoms to death ranged from 6 to 41 days with a medium of 14 days [1]. This time period is dependent on the patient's immune system and age. The incubation period was shorter among patients over 70 years of age and with pre‐existing medical conditions such as asthma, hypertension or diabetes [18].

A large proportion of the patients show mild symptoms and recover on their own. About 22% of the patients develop severe symptoms, and the incidence of critically severe illness was about 10·5% [15]. Another study reported that about 16% of the patients admitted to the intensive care unit (ICU) [19]. The most common symptoms at the onset of infection are malaise, dry cough and high fever as well as other symptoms including lymphopenia, diarrhoea, haemoptysis, headache, chills, repeated shaking with chills, muscle pain, sore throat and loss of taste or smell [18]. Once developed into severe pneumonia, COVID‐19 patients can have ARDS and changes in heart and liver function as a secondary or related consequence of disease, which could lead to multiple organ failures and death. These aforementioned severe symptoms may stay weeks in such patients.

Taken together, the time period of COVID‐19 symptoms can be approximately divided into three phases: (a) early infection phase: fever, dry cough, diarrhoea and headache; (b) pulmonary phase: shortness of breath and hypoxia; and (c) hyperinflammation phase: ARDS, shock and MODS [20].

### SARS‐CoV‐2 structure, viral cycle and ACE2 receptors

Belonging to Coronaviridae family, SARS‐CoV‐2 is enveloped non‐segmented, single‐stranded, positive‐sense RNA virus. So far, coronaviruses are the largest known RNA viruses. The viral particles display a rough spherical or multi‐faceted crystal shape. The surface has prominent club‐shaped projections composed of its structural protein and inside is the viral genome wrapped in a nucleocapsid [21, 22]. Viral genomes contain a 5’ cap and 3’ poly(A) tail. Approximately one‐thirds of the 3’ end RNA sequence encodes four core structural proteins, including spike (S), envelope (E), membrane (M), and nucleocapsid (N) proteins. Approximately two‐thirds of the viral genome capacity (the 5’ cap) is composed of ORF1a and ORF1b and encodes nonstructural replicase/transcriptase [21, 22]. S protein is a trimeric glycoprotein with S1 and S2 functional domains. S1 together with the receptor‐binding domain initiates the viral entry. S2, containing amino acid sequences of viral infectivity, induces the fusion between cytomembrane and viral membranes during endocytosis. E protein is a non‐glycosylated transmembrane protein found in small quantities, which facilitates the assembly and the budding process. M protein is the most abundant membrane protein on the viral particle, which contributes to the shaping and maturation of the virion. N protein, containing a C‐terminal domain and an N‐terminal domain, participates in RNA genome encapsulation and replication via direct binding with viral RNA [23]. Besides the four main structural proteins, there are sixteen nonstructural proteins (NSP) in SARS‐CoV‐2. They display diverse functions majorly in regulating viral replication–transcription machinery [24].

SARS‐CoV‐2 enters the host alveoli via the respiratory tract. Inside the alveoli, SARS‐CoV‐2 infection is robust in type II pneumocytes expressing angiotensin‐converting enzyme 2 (ACE2) receptor, a type I integral membrane protein of renin–angiotensin systems that control cardiac and kidney functions [25, 26]. Growing evidence has pointed out ACE2 as the pivot receptor for SARS‐CoV‐2 infection [26]. Both SARS‐CoV (glutamine 479 in the receptor‐binding domain) and SARS‐CoV‐2 (glutamine 394 in the receptor‐binding domain) can bind to lysine 31 on the human ACE2 with high affinity [27]. In addition, SARS‐CoV‐2 may be more efficient than SARS‐CoV in the perspective of human ACE2 recognition, increasing the spreading ability of SARS‐CoV‐2 among people.[27] The interaction of trimeric SARS‐CoV‐2 fusion protein (the S1 domain of S protein) with the peptidase domain (PD) of ACE2 recruits transmembrane protease serine 2 (TMPRSS2) to cleave the S1/S2 site of ACE2 (C‐terminal segment residues 697 to 716), thus enhancing viral entry [25]. Once SARS‐CoV‐2 is endocytosed into cell cytoplasm, its lipid bilayers are disassembled by lysosomal enzymes of type II pneumocytes. Afterwards, SARS‐CoV‐2 utilizes host cell RNA polymerase to replicate viral single‐stranded RNA, increasing the viral load within the host cell. Ribosomes of the host cell are also used to translate viral RNA into structural framework polyproteins [28]. These polyproteins can use host enzymes, such as proteinases, to further proteolyse themselves into S, E, M, and N proteins [28]. Structural proteins, together with viral RNAs, form mature SARS‐CoV‐2 that buds off the type II pneumocyte to get into the alveolus. This forms a vicious circle and aggravates the course of COVID‐19.

Besides type II alveolar epithelial cells, single‐cell RNA‐sequencing analyses also identified the co‐expression of ACE2 and TMPRSS2 in cardiomyocytes, vascular smooth muscle cells, renal tubular and intestinal epithelial cells [29]. The high expression of ACE2 augments SARS‐CoV‐2 infection in the lung, heart and small intestine that explains the pathophysiology of acute lung and myocardial injury, and gastrointestinal symptoms reported in COVID‐19 cases.

### Acute inflammatory response and cytokine storm

The budding off of SARS‐CoV‐2 leads to the destruction of host cells like the type II pneumocytes, which induces the release of various inflammatory mediators that stimulate alveolar macrophages. Activated macrophages release pro‐inflammatory cytokines including interleukin 1 (IL‐1), interleukin 6 (IL‐6) and tumor necrosis factor‐alpha (TNF‐α) [12, 30]. After entering the vascular system, these cytokines cause smooth muscle dilation and blood vessel endothelial cell contraction, collectively increasing capillary permeability [31]. As a consequence, plasma from the blood vessel leaks into the interstitial spaces and causes alveolar oedema. The rapid increase of blood cytokines and chemokines also attracts many inflammatory cells, such as neutrophils and monocytes, to lung tissue, resulting in inflammatory cells influx. Through stimulation of interferon (IFN) receptors, the accumulating inflammatory cells produce monocyte chemokines (e.g., CCL2, CCL7, CCL12) and pro‐inflammatory cytokines (e.g., TNF, IL‐6, IL‐1β), leading to further accumulation of pathogenic inflammatory cells [32]. While fighting viral pathogens, the excessive production of pro‐inflammatory cytokines and chemokines by neutrophils and monocytes in patients’ lung tissues and peripheral blood forms cytokine storm to attack more healthy cells [30]. The mass damage of type I and II pneumocytes also reduces the production of surfactant, which increases the surface tension within alveolus, contributing to alveolar collapse and alveolar oedema [31]. This alveolar collapse impairs gas exchange and leads to refractory hypoxemia. Peripheral chemoreceptors, triggered by hypoxemia, cause the sympathetic nervous system to increase respiration and heart rate to compensate the shortened gas exchange. With decreased partial pressure of oxygen, patient's breathing becomes increasingly difficult, resulting in ARDS.

Moreover, vascular cytokines can reach the central nervous system, especially the hypothalamus that is responsible for maintaining body temperature. The excessive IL‐1 and IL‐6 within the hypothalamus increase the production of prostaglandins that elevates the core body temperature to initiate fever. Excess cytokines that circulate through the vascular system can also be taken up by other tissue and trigger systemic inflammatory response syndrome (SIRS) [12, 30]. Vascular cytokine‐mediated capillary hyperpermeability induces plasma to deposits within tissues other than lung, therefore decreasing the blood volume. The vasodilation decreases the total peripheral resistance and reduces blood pressure significantly, which results in exhausted perfusion and ultimately leads to multi‐system organ failure (MSOF) [30]. The decreased perfusion to kidneys increases the blood urea nitrogen (BUN) and creatinine, leading to acute renal injury. Besides ARDS and MSOF, disseminated intravascular coagulation (DIC) has been identified as a severe complication in some COVID‐19 cases and an intermediate segment in the development of MSOF [33].

## THE BIOGENESIS, COMPOSITION AND ORIGINS OF EVS IN THE LUNG

Emerging evidence implicates EVs as an important intercellular communicator [4]. EVs are detected in cell culture supernatants and in virtually all biological fluids such as blood, sputum, bronchoalveolar lavage fluid (BALF), urine, cerebral spinal fluid, breastmilk and ascites [2]. As one of the hottest topics of the basic and clinical research, the biogenesis, composition and origins of EVs are under comprehensive investigation, leading to rapid expansion of our knowledge in disciplines aforementioned.

### The biogenesis of EVs

**FIGURE 1:**
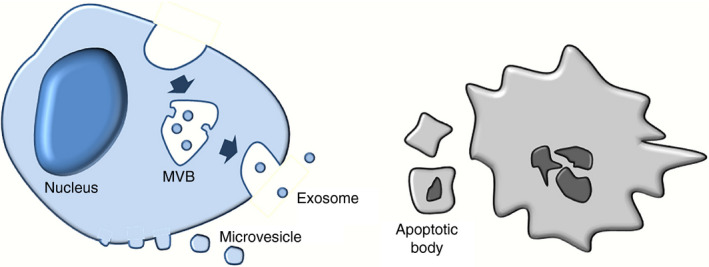
The biogenesis of extracellular vesicles (EVs). EVs contain three main types, exosomes, microvesicles (MVs), and apoptotic bodies. The biogenesis of exosomes initiates from the endocytosis of plasma membrane and the inward budding of endosomal membranes to from multiple vesicular bodies (MVBs). Exosomes are then released into extracellular space after the fusion of MVB with cell membrane. MVs are generated by the outward budding of cell membrane. Apoptotic bodies are formed by membrane‐blebbing of cells undergoing apoptosis.

Based on their biological characteristics, EVs are generally separated into three main classes including exosomes, microvesicles (MVs) and apoptotic bodies (Figure 1) [34, 35].

Exosomes are the smallest type of EVs (30–150 nm) and are characterized by their endosomal origin [2, 36]. The biogenesis of exosomes initiates from the formation of early endosomes (EEs). EEs mature into late endosomes that are also known as multivesicular bodies (MVBs). MVBs’ membrane buds inwardly to form intraluminal vesicles (ILVs). Then, MVBs can be guided to lysosomes for degradation or fuse with plasma membrane under the regulation of Rab GTPases and SNARE proteins. ILVs that are released into extracellular milieu via secretory pathway are referred to as exosomes. The unique origin of exosomes determines that exosomes are enriched with endosomal markers, including tetraspanins (CD9, CD63, CD81 and CD82), heat‐shock proteins (Hsc70 and Hsp90), ALG‐2‐interacting protein X (Alix), tumour susceptibility gene 101 (Tsg101) and major histocompatibility complex (MHC) classes I and II [34].

MVs (previously named as microparticles) have sizes between 100 and 2000 nm in diameter and are generated via direct outward budding of the plasma membrane [34]. MVs contain membrane components that are similar to that of the parent cell membrane‐like selectins, integrins, CD40 ligand, flotillin‐2 and adenosine diphosphate ribosylation factor 6 (ARF6) [37]. Although the biogenesis of EVs is not fully understood, reports claimed that MVs may be released under stimulation, while exosomes are generally secreted in a continuous manner [38, 39, 40].

Apoptotic bodies are the largest class of EVs with sizes of 1–4 μm in diameter [34]. Unlike exosomes and MVs that are secreted by cells under various conditions, apoptotic bodies are only released by cells undergoing apoptosis [34]. Apoptotic bodies are produced by cell membrane‐blebbing during the systematic breakdown of apoptotic cells. Thus, the biogenesis of apoptotic bodies may share secretory pathways with MVs, but only apoptotic bodies contain fragmented nucleus and organelles [34]. It is worth noting that the majority of functional studies of EVs exclude apoptotic bodies, as they neither reflect most physiological and pathological status states of parent cells, nor undertake normal intercellular communication [4, 35].

After being released into the microenvironment, EVs fuse with the plasma membrane of target cells directly, be internalized by recipient cells via endocytosis or phagocytosis or bind to target cells through ligand–receptor interactions [2, 34]. These vesicles therefore mediate cell‐to‐cell communication by transferring bioactive molecules into recipient cells or regulating the downstream signal cascades of activated receptors on target cells.

### The composition of EVs

EVs contain multiple types of functionally relevant biomolecules including proteins and peptides (e.g., endosome‐associated proteins, membrane proteins and lipid raft proteins), nucleic acids (e.g., DNA, mRNA and non‐coding RNA) and lipids. The contents of EVs vary with the origins and the pathophysiological states of parent cells. For example, although both lung epithelial cells and macrophages secret EVs enriched with cytokines and other inflammatory proteins and microRNAs (miRNAs) [41], EVs derived from both types of cells can be separated by the surface proteins of lung epithelial cells (e.g., pulmonary surfactant proteins and caveolin‐1) and macrophages (e.g., Ly‐6G/Ly‐6C) [42]. Similarly, EVs derived from other types of lung cells can also be identified by their unique surface markers. Besides, distinct membrane proteins are also present on exosomes and MVs. Transferrin receptors are highly enriched in exosomes, while vesicle‐associated membrane protein 3 (Vamp3) is predominantly expressed in the MVs [34]. Therefore, even though exosomes and MVs are similar in density and size that make it difficult to separate them by ultracentrifugation, the current gold standard for EV purification, different types of EVs released by various cells can be distinguished by detecting specific surface markers.

### The origins and types of EVs in the lung

To only understand the function of mixed exosome population in biological fluids, no longer satisfies the requirement of current research. It is important to figure out the origins of EVs and investigate the roles of cell‐specific EVs under various physiological and pathological conditions. In the perspectives of lung disease diagnosis and their pathogenesis research, blood (plasma or serum) and BALF are the most commonly used ones for EV isolation. As one of the easiest accessible biological fluids, plasma/serum is enriched with EVs. However, the origins of blood EVs are difficult to trace since EVs can be released by virtually all tissues and cells in the human body. Thus, BALF is a better source for isolating lung cell‐derived EVs.

With the help of the surface marker screen mentioned above, the origins of BALF EVs can be distinguished. Mounting studies demonstrated that pulmonary EVs can be released from many types of cells, including but not limited to lung epithelial cells, endothelial cells, alveolar macrophages, neutrophils and lymphocytes fibroblasts. Among them, epithelial cells and macrophages are the two main contributors of BALF EVs [43]. More importantly, mounting studies have revealed that the cellular origins of EVs vary in response to different pathological stimulations. In normal conditions without noxious stimuli, alveolar macrophages are the major source of BALF EVs [44]. Sterile stimuli, such as the exposure of oxidative stress or acid inhalation, cause diffuse alveolar cell damage. The perturbation of cell homeostasis induces robust EVs release from lung epithelial cells (e.g., alveolar type‐I epithelial cells) but not other types of cells in the lung. Thus, the majority of the BALF EVs are derived from lung epithelial cells under sterile stimuli till lung injury induces strong inflammatory response [42]. In contrast, infectious stimuli, such as bacterial and viral infection, activate alveolar macrophages, the first responder to infection, to trigger extensive pro‐inflammatory responses. Once activated, the EV generation capacity of macrophages increases dramatically. Alveolar macrophages, therefore, act as the main contributor to BALF EVs [42].

Interestingly, the type of noxious stimuli and the severity of diseases also influence the type of EVs detected in BALF. Multiple independent groups observed that MVs are the dominant type of EVs in BALF after hyperoxia or acid exposure [44, 45]. Both nanotracking analysis (NTA) and western blotting results suggested that MVs contribute to more than half of BALF EVs, especially in the early phase of sterile stimuli exposure. This situation also applies to lipopolysaccharide (LPS)‐induced pulmonary inflammation [46].

Taken together, EVs in BALF and other biological fluids are highly heterogeneous and dynamic. With the rapid development of technologies in high‐throughput screening and nanoscale particle separation and analysis, the types of EVs and their origins in BALF can be clarified. This important information, together with the functional studies, greatly promotes relevant research aiming to unveil the involvement of EVs in the pathogenesis of lung diseases, in particular COVID‐19.

## THE ROLES OF EVS IN SARS‐COV‐2 INFECTION

The contribution of EVs to infectious disease including COVID‐19 is a newly developed topic. For example, antimalarial drugs, such as chloroquine (CQ) and its analogue hydroxychloroquine (HCQ), have been found to exhibit antiviral effects against SARS‐CoV‐2 via blocking EV release, endocytosis and phagolysosomal fusion in vitro [47, 48]. Those in vitro studies have indicated EVs as a key mediator of SARS‐CoV‐19 infection, although following clinical trials have implied that CQ or HCQ may not bring prominent benefits to COVID‐19 patients but cause potential harm [49].

**FIGURE 2:**
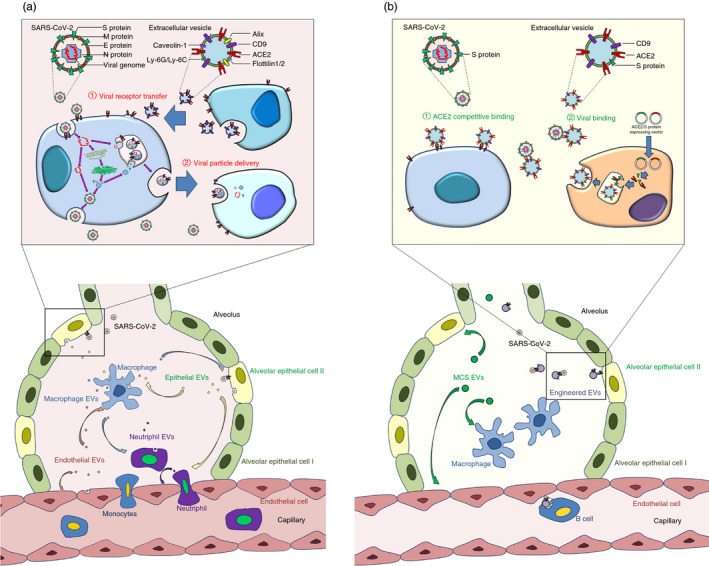
Roles of extracellular vesicles (EVs) in COVID‐19. (a) The pathological roles of EVs in COVID‐19. EVs transfer viral receptors ACE2 and CD9 among lung cells, making cells more susceptible to SARS‐CoV‐2 infection. Infected epithelial cells release EVs to enhance microphage activation and immune cell infiltration. Stimulated macrophages secrete EVs cause epithelial damage and accelerate neutrophil influx. Endothelial cells and recruited neutrophils generate EVs to increase capillary permeability and cytokine release from inflammatory cells, ultimately resulting in cytokine storm and acute lung injury. The detailed information of the EV‐based delivery of viral receptors/particles is provided on the top of the panel. (b) The therapeutic roles of EVs in COVID‐19. Mesenchymal stromal/stem cell‐derived EVs (MSC‐EVs) decrease inflammatory cell influx, block cytokines accumulation in the lung, elevate intracellular ATP levels and reduce oxidative stress, thus attenuating exudative pneumonia. EVs can be further equipped with viral proteins and receptors, leading to the production of neutralizing antibody in B cells, the competitive occupation of viral receptors on cell surface and the direct binding of virus to prevent the virus–host interaction. The detailed information of EVs engineering for viral binding and blocking viral receptors on cell membrane is provided on the top of the panel.

Current studies have revealed two main mechanisms for EV‐mediated viral infection (Figure 2a). First, EVs carry host proteins that make recipient cells more susceptible to SARS‐CoV‐2 infection. The infection of SARS‐CoV‐2 requires multiple steps including ACE2‐mediated receptor‐binding and TMPRSS2‐mediated intracellular cleavage. Recent studies identified ACE2 in EVs and demonstrated the transfer of ACE2 among various types of cells via EVs [50]. It implies that SARS‐CoV‐2 may utilize a similar strategy to human immunodeficiency virus (HIV), another type of RNA virus, regarding to virus internalization, in which SARS‐CoV‐2 enters target cells via binding to exosomal ACE2 [50]. Moreover, this finding has inspired a competitive inhibition therapy against SARS‐CoV‐2, which uses ACE2‐expressing EVs to occupy SARS‐CoV‐2 S protein S1 domain in a competitive manner, therefore protecting ACE2‐expressing cells from viral infection [51]. Another mechanism for EV‐mediated viral entry involves one of the most abundantly expressed proteins on the surface of EVs, tetraspanin CD9 [2]. It is reported that CD9 and TMPRSS2 work together in cleaving viral fusion glycoproteins and facilitate a quick entry coronavirus (e.g., MERS‐CoV) into lung cells [52]. Besides, CD9 also accelerates lentiviral infection and enhances transduction efficiency in immune responsible cells including B cells and T lymphocytes [53]. These observations reveal that CD9 and other tetraspanins on exosomal surface may be a mediator in SARS‐CoV‐2 infection. Besides ACE2 and tetraspanins, other host proteins on EVs also participate in infection. For example, coronavirus employs caveolin‐1‐dependent endocytosis for cell entry [54]. This entry is dynamin‐dependent, which requires actin cytoskeleton rearrangements. Since caveolin‐1 is present in lung epithelial cells and EVs are derived from these cells, EVs caveolin‐1 may play a supportive role for SARS‐CoV‐2 infection by spreading this protein in vivo.

Second, EVs may mediate the spreading of SARS‐CoV‐2 particles or components directly. Previous studies have demonstrated that circulating exosomes isolated from lung transplant recipients diagnosed with the infection of respiratory viruses including rhinovirus and respiratory syncytial virus contained viral antigens [55]. Additionally, by the ectopic expression in EV‐secreting cells (e.g., T293 cells), S protein can be loaded into EVs, which facilitates the interaction of SARS‐CoV‐2 with target cells [56]. These observations imply that SARS‐CoV‐2‐infected cells produce EVs containing virus particles, which accelerate the spreading of virus or exacerbate the host immune response, leading to cytokine storm. This premise is confirmed by multiple studies. For example, through transducing the selected genes of the SARS‐CoV‐2 into lung epithelial cells, EVs derived from transduced cells contain viral RNA [57]. Cardiomyocytes are able to internalize these EVs and take in these viral contents, which subsequently leads to an up‐regulation of inflammation‐related genes [57].

Although our knowledge about the roles of EVs in SARS‐CoV‐2 infection remains limited, numerous studies on RNA virus in general and coronavirus in particular offer important prompts in decrypting the molecular mechanisms involved in SARS‐CoV‐2 infection and spreading, and further suggesting directions in developing effective therapeutic strategies.

## THE PATHOLOGICAL ROLES OF EVS IN CYTOKINE STORM

As we described above, the infection of SARS‐CoV‐2 per se is not fatal, and the cytokine storm‐mediated ARDS and other complications are the main causes of death in COVID‐19. Therefore, the investigation of EVs’ involvement in cytokine storm has emerged as an essential research field.

The studies that explore the pro‐inflammatory roles of EVs in the lung started from the quantification of EVs among donors with cytokine storm/ARDS and their corresponding controls. It is reported that higher concentrations of MVs were detected in pulmonary oedema fluid (PEF) collected from patients with ARDS, compared with a control group of patients with hydrostatic pulmonary oedema [58]. Following studies demonstrated that EVs in BALF derived from LPS‐induced cytokine storm models were able to initiate inflammatory responses in the lung. These EVs were taken by mouse epithelial cells MLE12 and enhanced the expression of TNF‐α, IL‐6 and junction proteins in the latter in vitro [59]. Another group showed that after being incubated with LPS‐treated BALF EVs, MLE12 expressed higher levels of ICAM‐1 and keratinocyte‐derived cytokine (KC) [46]. More importantly, in LPS‐induced mouse lung inflammation model, the administration of GW4869 significantly decreased lung elastance, reduced the production of pro‐inflammatory cytokines and impaired alveolar collapse [60]. Similar results were obtained when applying GW4869 in asthmatic inflammatory conditions [43]. These functional studies demonstrate great potential of EVs in initiating and aggravating cytokine storm. Furthermore, the pro‐inflammatory effects of EVs derived from individual types of lung cells have been investigated.

### Epithelial cell‐derived EVs and cytokine storm

Alveolar epithelial cells are suggested as the entry points of SARS‐CoV‐2 in the lung. Bastarache et al. found that ARDS patients’ PEF‐EVs are likely originated from alveolar epithelial cells, implying the contribution of epithelial cell‐derived EVs in cytokine storm and ARDS. Similar results were acquired by Soni et al. that epithelial cells are the main contributors of EVs in BALF 4 h after LPS intratracheal instillation (i.t.). With LPS stimulation, airway epithelial cells release excessive EVs that are enriched with prolyl endopeptidase (PE), an extracellular protease critical to the regulation of inflammatory responses in various human chronic lung diseases [61]. When treated with pro‐inflammatory cytokines (TNF‐α, IL‐1β, IFN‐γ), alveolar epithelial cells secrete abundant MVs containing procoagulant tissue factor (TF), which participates in fibrin deposition in ARDS [58]. The mechanisms of epithelial cell‐derived EV‐mediated inflammatory response have been explored. Under oxidative stress and acid inhalation, epithelial cell‐derived EVs can be internalized by alveolar macrophages and enhance the production of TNF‐α, IL‐6 and macrophage inflammatory protein 2 (MIP‐2) in the latter [62]. These EVs also robustly increase macrophage and neutrophil influxes in lung tissue [62]. Epithelial cell‐derived EVs transfer certain miRNAs (miR‐221 and miR‐320a) into macrophages and in this way upregulate the integrin β1‐mediated macrophage/neutrophil extravasation and migration into inflammation sites [62]. Unlike the situation of sterile stimuli, the effects of epithelial cell‐derived EVs under infectious stimuli remain largely unknown. This knowledge gap needs to be filled urgently in order to fight against SARS‐CoV‐2‐induced cytokine storm.

### Macrophage‐derived EVs and cytokine storm

Macrophages are the first responders to viral infection among all immunoregulatory cells and are therefore strongly associated with cytokine storm in the lung. We, together with others, have demonstrated that macrophages secrete large amounts of EVs when treated with LPS or infected with viruses [41, 63]. The characterization analyses of EVs showed that the majority of MVs in BALF of mouse challenged with i.t. LPS for 1 h are derived from alveolar macrophages [46]. In vitro studies suggest that MVs released from LPS‐primed alveolar macrophages induce immune response of epithelial cells including the expression of the inflammatory protein ICAM‐1 [46]. Post‐intravenous administration, LPS‐stimulated alveolar macrophage‐derived EVs cause cytokine storm‐like phenotypes including the elevation of pro‐inflammatory cytokine levels, the infiltration of immune cells, haemorrhage, interstitial and alveolar oedema and the thickness of alveolar septum [8]. Composition analyses identified that various pro‐inflammatory factors, such as TNF‐α and glutaminase (GLS), are massively packaged into these EVs [46, 63]. TNF‐α in macrophage‐derived EVs activates NF‐κB in lung epithelial cells, thus leading to the overproduction of interleukin 8 (IL‐8) and monocyte chemoattractant protein 1 (MCP1) [64]. IL‐8 and MCP1 attract neutrophils and monocytes to pulmonary tissue, which, in turn, causes cytokine storm. GLS, delivered by EVs, can also activate macrophages via dysregulating cellular metabolic activities, such as the abnormal accumulation of reactive oxygen species (ROS) [41, 63, 65]. Besides, multiple pro‐inflammatory miRNAs are also enriched in EVs originated from LPS‐primed macrophages/microglia [41, 66, 67]. Thus, although we are still in lack of evidence for the roles of macrophage‐derived EVs in COVID‐19 cytokine storm, data obtained in LPS‐stimulated model indicate these EVs as a key factor in lung inflammation and injury.

### Neutrophil‐derived EVs and cytokine storm

Except for macrophages, neutrophils are important mediators in inflammatory responses. Neutrophil influx into pulmonary tissue has been regarded as a key step of cytokine storm. As we described above, many studies have focused on the effects of EVs originated from epithelial cells or macrophages on the infiltration of neutrophils, but the function of neutrophil‐derived EVs is largely unknown. In 2019, Genschmer et al. demonstrated that neutrophil activated by bacterial formylated peptide (a canonical PMN stimulant) formyl‐methionine‐leucine‐phenylalanine (fMLP) released EVs with high neutrophil elastase (NE) activity [68]. These EVs caused emphysema when administered into murine lungs via NE‐mediated degradation of extracellular matrix (ECM). Furthermore, Rossaint et al.[69] found that platelets interacted with intravascular neutrophils through P‐selectin/P‐selectin glycoprotein ligand 1 (PSGL‐1)‐mediated binding and enhanced the generation of EVs from the latter. Neutrophil‐derived EVs induce the synthesis of thromboxane A2 (TxA2) in platelets, which further increases endothelial ICAM‐1 expression. The presence of endothelial ICAM‐1 recruits neutrophils, intensifying inflammatory responses of the lung. Additionally, EVs derived from LPS‐stimulated neutrophils can enhance the proliferation of airway smooth muscle cells (ASMCs). The excessive generation of ASMCs thickens airway wall and contributes to lung inflammation [70]. These studies indicate that neutrophil‐derived EVs may be widely associated with COVID‐19 cytokine storm by facilitating neutrophil infiltration, inducing lung injury and developing exuberant inflammation.

### Endothelial cell‐derived EVs and cytokine storm

Endothelial cells are another type of cells associated with cytokine storm and ARDS. Endothelial injury can cause severe sequelae of pulmonary capillary leak, microvascular thrombosis and physiologic shunt. Endothelial cells release EVs robustly under cytokine stimulation, starvation, mechanical ventilation or cigarette smoke [71, 72]. These EVs cause attenuation of endothelium‐mediated vasodilation and a significant rise in pulmonary capillary permeability by smothering NO release [71]. The compromise of the endothelial–alveolar barrier recruits neutrophil and triggers strong inflammatory response. Endothelial cell‐derived EVs can also be internalized by macrophages, leading to the dysfunction of the latter [72].

Besides aforementioned cell types, EVs derived from other cells such as red blood cells are also involved in neutrophil priming and other key steps of the development of cytokine storm [73]. Although the exact roles of EVs in COVID‐19 cytokine storm remain largely unknown, current findings suggest that the excessive production of pro‐inflammatory EVs is an indispensable link in the chain reaction of cytokine storm, implying EVs as a promising therapeutic target in treating cytokine storms in COVID‐19.

## THE POTENTIAL APPLICATION OF EVS IN COVID‐19 DIAGNOSIS AND PROGNOSIS

Growing studies implicate cargos in biological fluid‐isolated EVs as potential biomarkers in the diagnosis and prognosis of diverse diseases [2]. The possibility for recruiting EVs in COVID‐19 diagnosis is also under meticulous examination. Goodlet et al. analysed the contents of serum EVs isolated from a COVID‐19 patient [74]. Before infection, no viral protein could be detected in circulating EVs, while SARS‐CoV‐2 S protein could be found in EVs after the patient contracted SARS‐CoV‐2. Post‐infectious symptom resolution, viral proteins could no longer be identified in serum EVs. This work indicates the feasibility of EV‐based COVID‐19 diagnosis, although more work remains to be done including the screening of viral molecules in a larger number of samples, the specificity/sensitivity validation of identified diagnostic index candidates and the development of simple and inexpensive EV cargo detection methods.

Furthermore, EVs in peripheral blood may also be used as prognostic markers for COVID‐19 [75]. COVID‐19‐induced endothelial cell damage promotes EV release and is also likely to contribute to prothrombotic environment [76, 77]. The release of endothelial cell‐derived EVs with surface‐bound coagulation factors (prothrombin and factors VII, IX and X) and von Willebrand factor (VWF) can result in COVID‐related deaths through venous thromboembolism. Since these EVs are only secreted from damaged endothelial cells to peripheral blood, they could be used for COVID‐19 severity classification, suggesting the clinical application of EVs in COVID‐19 prognosis in treatment.

## THE THERAPEUTIC EFFECTS OF EVS IN TREATING COVID‐19

EVs are emerging as a potential alternative of the whole cell‐based therapy. The advantages of EVs include ease of access, lower risk of tumorigenesis and possibility for long‐term storage [2]. More importantly, EVs possess bilayer phospholipid membrane, which protects their contents from degradation and facilitates their fusing with target cells. To date, many animal studies and clinical trials have been carried out to examine the therapeutic effects of EVs in human diseases. Inspiringly, by modifying the origins, composition and administration approaches, EVs achieve promising treatment outcome in various lung diseases including COVID‐19 (Figure 2b) [78].

### Stem cell‐derived EVs as promising medications for treating COVID‐19

Now in the field of COVID‐19 therapeutic strategy development, EVs derived from stem cells, especially mesenchymal stromal/stem cells (MSCs), attracted great attention [79]. MSCs are multipotent cells that play an irreplaceable role in the pathogenesis and recovery of multiple inflammatory diseases, with their immunosuppressive and anti‐inflammatory properties [80]. A great number of studies have demonstrated the promising therapeutic outcome of MSC‐derived EVs (MSC‐EVs) treatment in various models of pneumonia. Therefore, MSC‐EVs have been considered as a promising candidate for therapeutics in combating COVID‐19 [79, 81]. Currently, multiple clinical trials have been registered on clinicaltrials.gov and at least one clinical trial was finished in which severe COVID‐19 patients with moderate‐to‐severe ARDS received a single 15 ml intravenous dose of ExoFlo™, a MSC‐derived exosome agent [82]. The clinical safety is confirmed. Patients’ clinical states and oxygenation were improved, accompanied with a decline of absolute neutrophil counts, a rise of lymphocyte counts and a reduction of acute phase reactants (e.g., C‐reactive protein, ferritin and D‐dimer) after one treatment.

The therapeutic effects of MSC‐EVs in COVID‐19 are highly likely mediated by transferring protective and anti‐inflammatory RNAs and proteins among damaged or activated cells in pulmonary tissue [2, 83, 84, 85]. MSC‐EVs are highly enriched with multiple miRNAs (e.g., let‐7 [83], miR‐124‐3p [2], miR‐21‐5p [84], miR‐146a [2] and miR‐145 [85]). miR‐124‐3p can repress oxidative stress and cytokine expression by directly binding to purinergic receptor P2X ligand‐gated ion channel 7 (P2X7) or regulating Toll‐like receptor‐related genes [2]. miR‐21‐5p suppresses lung cell apoptosis via inhibiting *PTEN* and *PDCD4* [84]. miR‐146a shifts the phenotypes of macrophage from pro‐inflammatory to anti‐inflammatory via repressing Nf‐κb signalling pathway [2]. miR‐145 significantly enhances the phagocytic capacity of macrophages to accelerate microbial clearance [85]. Therefore, although EV‐based therapy is a new topic, current knowledge has implicated MSC‐EVs as a promising ‘medicine’ in treating COVID‐19 through the manipulation of the complicated immunomodulatory network. Substantial efforts are now warranted to ensure the robustness and reliability.

Besides MSC‐EVs, EVs derived from other sources also exhibit potentials in treating ARDS. For instance, unstimulated neutrophil‐derived EVs play an anti‐inflammatory role on alveolar epithelial cells through miR‐223‐mediated PARP‐1 inhibition during acute lung injury [86]. Our understanding for the therapeutic potential of these EVs remain extremely narrow, comprehensive investigation, therefore, is required before we come to a definite conclusion.

### Engineered EVs as potential drugs for treating COVID‐19

Moreover, EVs derived from specific types of immune cells or equipped with viral or antiviral components may be used as therapeutic drugs to treat COVID‐19 directly. For example, a currently active clinical trial (ClinicalTrials.gov Identifier: NCT04389385) plans to use SARS‐CoV‐2 specific fragment peptide‐activated T‐cell‐derived EVs to treat early‐stage COVID‐19. These EVs may contain potent mediators including IFN‐γ that control disease progression effectively.

Besides, EVs may hinder SARS‐CoV‐2 infection via directly interacting with viruses or protecting susceptible cells from viral recognition [51, 87]. Since recombinant soluble ACE2 proteins can block SARS‐CoV‐2 infection by competitively inhibiting the binding of SARS‐CoV‐2 to ACE2‐expressing cells in vitro [88, 89], EVs equipped with ACE2 may limit the progression of viral infection in vivo [51, 87]. This scenario has been partially proved by Coffey's group [90]. They observed that EVs containing ACE2 bound SARS‐CoV‐2 through the virus S protein, indicating engineered EVs as a promising therapy for blocking SARS‐CoV‐2 infection. Another approach, reported by O'Driscoll et al., suggests that MSC‐EVs decorated with S proteins can occupy ACE2 on alveolar type II cells, competing against SARS‐CoV‐2 for cellular uptake and protecting cells from viral infection [91]. Therefore, many EV‐based therapies have been proposed or studied for treating COVID‐19. With comprehensive investigations, engineered EVs can play a huge role in the combat against COVID‐19 and probably other infectious diseases.

### Engineered EVs as potential vaccine for preventing COVID‐19

Besides therapeutics, vaccines that control SARS‐CoV‐2 are also urgently needed due to the current COVID‐19 pandemic [92]. Currently, multiple clinical trials that utilize lipid nanoparticles encapsulated with mRNAs (e.g., mRNA‐1273, BNT162b1, CVnCoV) and saRNAs (e.g., LNP‐nCoVsaRNA) are already carried out in Germany, Belgium and the United States [93]. Being natural nanoscale vesicles with lipid bilayer membrane, EVs are also contemplated as new and novel avenues for vaccine development [94, 95]. EVs interact extensively with immune cells and activate the latter, thereby priming the immune responses to recognize and kill specific types of cells [96]. For example, EVs isolated from malignant effusions of three patients with papillary adenocarcinoma kill tumour cells by inducing dendritic cells to prime T lymphocytes via a MHC I‐dependent mechanism [97]. Furthermore, Kuate and colleagues generated SARS‐CoV S protein‐decorated EVs that facilitate the B‐cell receptor cross‐linking and elevate neutralizing antibody titres in mice to a level higher than that in the convalescent serum of SARS patients [56]. This finding implies that EVs containing SARS‐CoV‐2 components may work as a vaccine for COVID‐19. Although we are in lack of evidence to consider EVs as SARS‐CoV‐2 vaccine, developing EV‐based nanoscale therapeutic vaccine is an important and interesting direction to go.

### EVs as drug delivery platform to treat COVID‐19

Growing studies have demonstrated EVs as promising natural carriers for drug loading and delivery due to multiple superiorities, such as high biocompatibility profile, long half‐life in vivo and the capability to cross biological barriers [81, 98, 99]. EV‐based delivery is utilized in treating various lung diseases. Post‐i.t., MyD88 siRNA‐ or miR‐223/142‐loaded EVs inhibit NF‐κb signalling pathway or Nlrp3 inflammasome activation, respectively, in alveolar macrophages, leading to the suppression of lung inflammation [67, 100]. In the perspective of COVID‐19 treatment, EV‐based drug delivery is also under heated discussion [47]. For instance, by modifying surface molecules, EVs can be conferred with the capacity to target SARS‐CoV‐2‐infected cells/tissues [99]. Homing molecules (e.g., nano/antibodies, DNA aptamer and peptides) that specifically target caveolin‐1 or Ly‐6G may guide EVs to lung epithelial cells or macrophages, respectively, and deliver anti‐inflammatory medicines to stifle cytokine storm in COVID‐19 [42, 99]. Other than the expression of cell‐specific ligands on the surface of EVs, the assembly of viral proteins that are involved in the targeting recognition onto EVs is another promising approach to achieve targeted delivery [101]. For example, EVs derived from SARS‐CoV‐2 model cell lines (e.g., Vero CCL‐81 or Vero E6) contain surface proteins that recognize SARS‐CoV‐2‐infected alveolar macrophages, implying that these EVs can deliver encapsulated drugs to the focus of infection [101]. Therefore, clinical trials for repurposing aforementioned drugs in COVID‐19 through EV‐based delivery can be very rapid, as these drugs are FDA‐approved and EVs are widely used natural nanovesicles in basic researches and clinical practices.

### Concerns for the translation of EVs usage in COVID‐19 from the bench to the bedside

As mentioned above, MSC‐EVs and engineered EVs both have great potential to treat COVID‐19. To date, multiple types of EVs have been recruited in clinical trials and numerous hypotheses of EV‐based therapies have been proposed. However, there are also concerns when the use of EVs is translated from the bench to the bedside.

The first one is unforeseen side‐effects that may be triggered by EVs due to our incomplete understanding on the mechanisms by which EVs exert their beneficial effects. For instance, adipose‐derived MSC‐EVs exhibit thrombogenic activity, which may result in catastrophic microvascular injury syndrome in severe COVID‐19 cases [102]. EVs from various types of immortalized cells including T293 cells may possess tumorigenic potential similar to cancer cells, which probably contribute to tumour microenvironment formation and cancer recurrence/metastasis [103]. Second, MSC‐EVs are reported to moderate acute immune responses towards regulatory responses that induce tolerance and restore homeostasis [104]. This tolerance induction may have severe adverse effects in the presence of replicating pathogens. Third, there is a lack of unified manufacturing and quality control for the clinical application of EVs. MSC‐EV preparations with comparable particle and protein contents can exhibit significant differences in therapeutic efficacy as not all preparations effectively protect neurons in a stroke model [105]. Fourth, although EVs are considered with low immunogenicity, we cannot exclude potential immunogenic and toxic risks of EVs in clinical practice. More seriously, we are currently in lack of the approaches to get rid of the harmful or unwanted cargos in EVs precisely due to technical limitations [106]. Thus, the translation of EVs usage from researches to clinical applications requires multiple steps including the collection of concrete pre‐clinical safety and efficacy data, the establishment of appropriate manufacturing and quality control provisions, the development of standardized clinical protocols and the minimization of risks and hazards.

Overall, although there are concerns for the usage of EVs, the aforementioned promising results give us confidence to consider EVs as potential therapeutics or delivery system. We believe that with the generation of appropriate manufacturing and quality control provisions, pre‐clinical safety and efficacy data, rational clinical trial design and proper regulatory oversight, the application of EVs in treating COVID‐19 and many other diseases is a near possibility.

## CONCLUSIONS

The involvement of EVs in lung diseases has been of great interest in the past several years. Being a key component of lung microenvironment, EVs derived from SARS‐CoV‐2‐infected cells may promote viral infection, replication, and spreading via delivering viral RNAs and proteins to healthy cells. These EVs also facilitate viral entry and escaping from immune cell recognition by equipping with SARS‐CoV‐2 receptors like CD9 and ACE2. Furthermore, EVs can modulate immune responses of alveolar macrophages and recruit more immune cells into the lung, leading to exuberant inflammation and cytokine storm. Except for the pathological roles of EVs, the pre‐clinical and clinical applications of EV‐based therapeutic strategies for treating COVID‐19 have been proposed. Through transferring anti‐inflammatory RNAs, MSC‐EVs decrease the infiltration of immune cells and block the accumulation of cytokines post‐i.t., protecting lung tissue from cytokine storm‐induced acute injury. EVs selectively loaded with anti‐inflammatory or antiviral agents may also demonstrate great therapeutic potential in treating COVID‐19. In addition, EVs decorated with viral receptors or proteins may block viral entry through binding with SARS‐CoV‐2 competitively or stimulating neutralizing antibody production, respectively.

In summary, numerous studies have demonstrated the tight association between EVs and the pathogenesis of coronavirus‐mediated pneumonia including COVID‐19. More comprehensive and meticulous research, aiming to fully unveil the pathological and therapeutic effects of EVs, will shed light on the development of EV‐based therapy.

## CONFLICT OF INTEREST

The authors declare no conflict of interests regarding the publication of this paper.
